# Expanded Umbilical Cord Blood Transplantation in Cerebral X‐Linked Adrenoleukodystrophy: A Case Report

**DOI:** 10.1002/jha2.70419

**Published:** 2026-09-28

**Authors:** Rachel E. Granberg, Thomas Pfeiffer, Sanil Sansar, Rafael Galindo, Shalini Shenoy

**Affiliations:** ^1^ Division of Hematology/Oncology Department of Pediatrics Washington University School of Medicine St. Louis Missouri USA; ^2^ Gamida Cell Therapeutics Naples Florida USA; ^3^ Division of Pediatric & Developmental Neurology Department of Neurology Washington University School of Medicine St. Louis Missouri USA

**Keywords:** case report, expanded cord blood, hematopoietic cell transplantation, metabolic disorders, umbilical cord blood transplantation, x‐linked adrenoleukodystrophy

## Abstract

Umbilical cord blood transplantation is a viable source of stem cells due to accessibility and low incidence of chronic graft‐versus‐host disease despite human‐leukocyte‐antigen mismatching. Disadvantages of low stem cell dose in larger recipients include delayed immune reconstitution, graft rejection, and mortality. Successful transplants with ex‐vivo expanded cords resulted in approval in malignant disorders and aplastic anemia. We report the first successful expanded cord transplantation in an adolescent with cerebral adrenoleukodystrophy who had no alternative donor/treatment options and required transplant emergently for neurologic preservation. This report highlights a mechanism for timely transplant in metabolic disorders, and preservation of functional advantages despite ex‐vivo expansion.

**Trial Registration**: The authors have confirmed clinical trial registration is not needed for this submission.

## 1 Background

Cerebral x‐linked adrenoleukodystrophy (CALD) is a rare genetic disorder caused by mutations in the *ABCD1* gene, leading to accumulation of very long chain fatty acids (VLCFAs), oxidative stress, neuroinflammation, and progressive loss of cerebral glia and myelinated axons, ultimately resulting in fatal neurologic decline. Allogeneic hematopoietic cell transplantation (HCT) can halt cellular degeneration, decrease inflammation, and preserve myelination by replacement of microglial cells [1]. Gene therapy (GT), although recently approved for use, is limited to younger patients with low neurologic functional scores (NFS) [2]. Barriers to HCT include carrier status in female family members and lack of suitable human‐leukocyte‐antigen (HLA) matched donors. Umbilical cord blood (UCB) allows more HLA mismatching due to lower incidence of graft‐versus‐host disease (GVHD) but is limited by cell dose. A minimum total nucleated count (TNC) of 3 × 10^7^/kg post‐thaw is recommended for UCBT in immune competent disorders to reduce graft rejection (GR), delayed engraftment, poor immune reconstitution, and infections. Higher cell doses in HLA‐mismatched products have significantly better outcomes at doses up to 6 × 10^7^/kg [3]. Ex‐vivo expanded UCB products can overcome cell dose limitations. Omisirge (Omidubicel) is the first such product approved for hematologic malignancies and aplastic anemia [4, 5]. We describe successful HCT with Omisirge in an adolescent with CALD ineligible for GT by age and without suitable donors.

## 2 Case Description

An 18‐year‐old male with scholastic and behavioral concerns was diagnosed with an *ABCD1* pathogenic mutation (c.901‐1G>A; Splice acceptor). His NFS was zero. Brain imaging demonstrated T2/FLAIR hyperintensities in the genu of the corpus callosum and bilateral frontal periventricular white matter with associated contrast enhancement in the genu consistent with the frontal variant of CALD and a Loes severity score of 4 (Figure 1). Frontal variant CALD is associated with poor neurologic outcomes despite HCT at Loes scores ≥ 4, unlike the classic variety where scores < 9 have favorable outcomes [1]. This unknown track of progression was extensively discussed with the patient and family in addition to the goals of stemming further deterioration. They favored proceeding with HCT.

**FIGURE 1:**
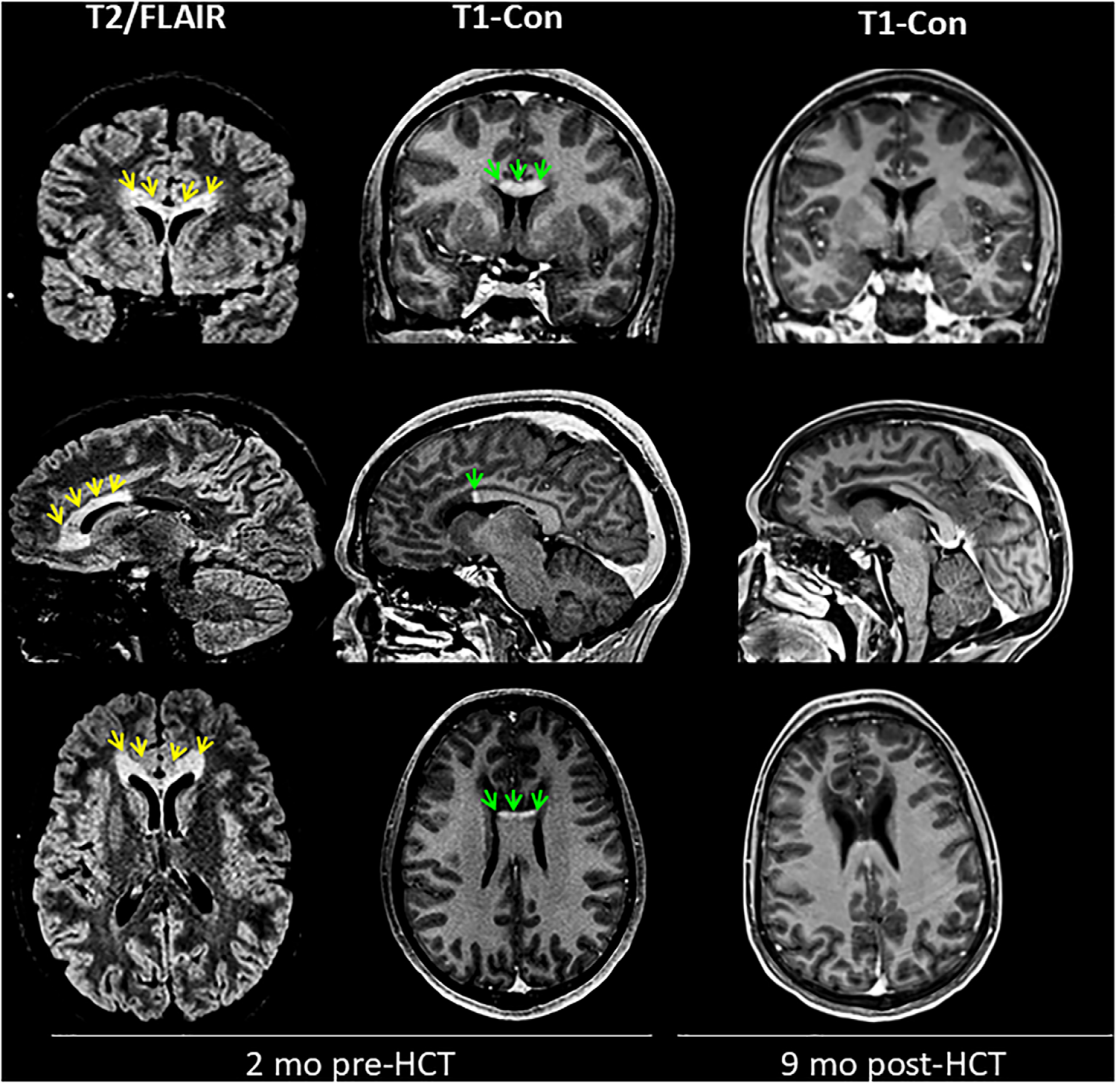
T2/FLAIR and T1 contrast (T1‐Con) coronal, sagittal, and axial MR sequences demonstrating T2/FLAIR hyperintensities involving the genu of the corpus callosum with associated atrophy as well as the bilateral frontal white matter (yellow arrows) accompanied by contrast enhancement (green arrows) followed by subsequent resolution of contrast enhancement 9 months post hematopoietic cell transplantation (HCT).

All available familial donors (mother and four siblings) carried the *ABCD1* mutation; no suitable registry donors were available. A single 8/10 HLA‐matched UCB unit (B and DPB1 allele mismatched) with low pre‐thaw TNC and CD34+ cell doses (3.64 × 10^7^/kg and 1.8 × 10^5^/kg, respectively) was identified. The low cell dose prompted compassionate use expansion by Gamida Cell (Kiryat Gat 82584120, Israel) [4, 5].

Hematopoietic stem cells were cultured with nicotinamide to inhibit differentiation and enhance function (homing/engraftment) [6]. The product included expanded CD133+ (cultured) and non‐expanded CD133− (non‐cultured) fractions. The final TNC, CD34+ and CD3+ cell doses infused were 37, 4.31, and 4.0 × 10^6^/kg respectively. The CD34+ cell dose infused was 24‐fold higher than the original product.

Following institutional review board approved consent, myeloablative conditioning administered included intravenous busulfan on Days −6 through −3 (target area under the curve 70–90 mg·h/L), fludarabine 30 mg/m^2^ every 24 h on Days −6 through −3, and thiotepa 5 mg/kg/dose every 12 h on Day −2. Planned GVHD prophylaxis included oral tacrolimus (target trough 8–12 ng/mL), mycophenolate mofetil 17 mg/kg thrice daily through Day +30, and intravenous abatacept 10 mg/kg/dose on Days −1, +5, +28, +60, and +100 per institutional practice. Abatacept doses were extended to include Days +128, +149, and +184 due to patient non‐compliance with oral tacrolimus [7].

Neutrophils (> 500/mm^3^) and platelets (> 20,000/mm^3^) engrafted on Days +16 and +56, respectively. Eltrombopag was administered between Days +18 and +38 for thrombocytopenia‐induced hematuria and gastrointestinal bleeding. Late acute Grade 1 (skin) and limited chronic GVHD (ocular) were diagnosed on Day +140. Skin biopsy demonstrated spongiotic dermatitis. Following complete resolution of GVHD (Day +211), tacrolimus was discontinued on Day +330.

Stable and full donor myeloid engraftment (> 95% donor CD15+ chimerism) was maintained at 3 and 12 months. Peroxisomal VLCFA (C26:0/C22:0) ratios were 0.042, 0.03, and 0.029 pre‐HCT and at 3 and 12 months post‐HCT, respectively (normal ≤ 0.023). At 18 months post‐HCT, he is employed (since 1‐year post‐HCT), neurologic examination is normal, all medications are discontinued, and immunizations resumed. Absolute CD4+ T‐cell counts were 141 and 252 cells/µL at 6 and 12 months, respectively. MRI (Days +100 and +265) showed near complete resolution of contrast enhancement at the anterior corpus callosum (Figure 1). The Loes score remains unchanged at four. Neurocognitive assessment (Day +79) identified mild attenuation of attention/executive functions, processing, reading, math, and writing speed. This involvement identified early posttransplant without acute HCT‐related events is consistent with the radiologic involvement noted from CALD and will be tracked over time. The NFS has remained zero. His Karnofsky performance score is 100.

## 3 Discussion

Adolescents with frontal variant CALD typically have rapidly progressive cognitive decline, prominent psychiatric manifestations, motor dysfunction, and cerebellar ataxia resulting in disability and death approximately 2 years after symptom onset [8]. Rarely spontaneous arrest of CALD lesions have been reported [9]. HCT remains the established standard of care for ALD with low Loes scores and NFS predicting better outcomes [1]. Successful donor myeloid engraftment correlates with improved gadolinium enhancement on MRI and disease stabilization, underscoring the importance of early HCT and robust myeloid reconstitution [10].

UCBT is a viable option for young patients with CALD in the absence of familial or suitably matched registry donors, offering rapid availability and less stringent HLA‐matching requirements. However, low cell dose and consequent GR remain challenges especially in older patients [3]. Cell dose limitations can be overcome with expanded UCB [11]. Omidubicel, a nicotinamide‐expanded UCB product, is the only approved expanded product for patients ≥ 12 years with hematologic malignancies and ≥ 6 years with severe aplastic anemia. Omidubicel demonstrated shorter time to engraftment, lower Grade 2–3 bacterial infections, reduced hospital stay, with comparable survival to other stem cell sources [5]. Omidubicel has been associated with increased GVHD as demonstrated in sickle cell disease patients [12]. Our experience suggests that this risk can be mitigated using abatacept without jeopardizing engraftment. A company sponsored study in non‐malignant disorders is under consideration.

This is the first report of successful HCT with expanded UCB in an adolescent with a metabolic disorder. GVHD risk was mitigated successfully by combining standard GVHD prophylaxis with abatacept and timely immune reconstitution facilitated re‐immunization. This approach merits investigation as a stem cell source for transplantation in metabolic disorders.

## Author Contributions

All authors contributed to writing and reviewing the manuscript.

## Funding

The authors have nothing to report.

## Ethics Statement

The current work was exempted from requiring ethics approval and consent to participate based on institutional guidelines.

## Consent

The patient in this report provided consent to publish these deidentified findings.

## Conflicts of Interest

Sanil Sansar is an employee of Gamida Cell. Shalini Shenoy was supported by Gamida Cell to present this case in part at the annual meeting of the Association for the Advancement of Blood and Biotherapies, October 2025. The other authors declare no conflicts of interest.

## Data Availability

Due to patient confidentiality and privacy protection, data on this patient are not publicly available. Additional information can be provided by the corresponding author upon reasonable request.
